# Apoptotic tumor cell-derived microparticles loading Napabucasin inhibit CSCs and synergistic immune therapy

**DOI:** 10.1186/s12951-023-01792-8

**Published:** 2023-02-02

**Authors:** Boping Jing, Feng Guo, Rui An, Yu Gao, Yuman Li, Yuji Xie, Jing Wang, Yihan Chen, He Li, Tang Gao, Qiaofeng Jin, Li Zhang, Mingxing Xie

**Affiliations:** 1 Department of Ultrasound Medicine, Union Hospital, Tongji Medical College, Huazhong University of Science and Technology, Wuhan, 430022 China; 2 Clinical Research Center for Medical Imaging in Hubei Province, 1277 Jiefang Ave, Wuhan, 430022 Hubei China; 3 Hubei Key Laboratory of Molecular Imaging, Wuhan, 430022 China; 4 Department of Pancreatic Surgery, Union Hospital, Tongji Medical College, Huazhong University of Science and Technology, Wuhan, 430022 China; 5 Department of Nuclear Medicine, Union Hospital, Tongji Medical College, Huazhong University of Science and Technology, Wuhan, 430022 China; 6 Shenzhen Huazhong University of Science and Technology Research Institute, Shenzhen, 518607 China

**Keywords:** Cancer stem cells, Napabucasin, Tumor-derived microparticles, PET/CT imaging, JAK-STAT pathway

## Abstract

**Background:**

Cancer stem cells (CSCs) are crucial for the growth, metastasis, drug resistance, recurrence, and spread of tumors. Napabucasin (NAP) could effectively inhibit CSC, but its mechanism has not been fully explained. Additionally, NAP also has the drawbacks of poor water solubility and low utilization. Therefore, this study not only elaborated the new mechanism of NAP inhibiting CSCs, but also built NAP-loaded nanoprobes using apoptotic tumor-derived microparticles (TMPs) as carriers to combine diagnose and treat of colon cancer and lessen the adverse effects of NAP.

**Results:**

The study discovered a new mechanism for NAP inhibiting tumors. NAP, in addition to inhibiting STAT3, may also inhibit STAT1, thereby inhibiting the expression of CD44, and the stemness of colon cancer. N_3_-TMPs@NAP was successfully synthesized, and it possessed a lipid bilayer with a particle size of 220.13 ± 4.52 nm, as well as strong tumor binding ability and anti-tumor effect in vitro. In static PET/CT imaging studies, the tumor was clearly visible and showed higher uptake after N_3_-TMPs@NAP injection than after oral administration. The average tumor volume and weight of the N_3_-TMPs@NAP group on day 14 of the treatment studies were computed to be 270.55 ± 107.59 mm^3^ and 0.30 ± 0.12 g, respectively. These values were significantly lower than those of the other groups. Additionally, N_3_-TMPs@NAP might prevent colon cancer from spreading to the liver. Furthermore, due to TMPs’ stimulation of innate immunity, N_3_-TMPs@NAP might stimulate anti-tumor.

**Conclusions:**

As a combined diagnostic and therapeutic nanoprobe, N_3_-TMPs@NAP could successfully conduct PET/CT imaging, suppress CSCs, and synergistically stimulate anticancer immune responses. Additionally, this nanoprobe might someday be employed in clinical situations because TMPs for it can be produced from human tissue and NAP has FDA approval.

**Supplementary Information:**

The online version contains supplementary material available at 10.1186/s12951-023-01792-8.

## Background

Accounting for nearly 10% of all cancer cases worldwide, colon cancer has become the third most prevalent cancer type with high mortality and morbidity [1, 2]. Current therapies for colon cancer are often ineffective due to the resistance of cancer stem cells (CSCs) to conventional chemotherapy and the initiation of tumor relapse and metastasis [3, 4]. The CSCs, also known as cancer-initiating cells, contribute to tumorigenesis and tumor metastasis, drug resistance, tumor recurrence, and dissemination [5]. Several studies have shown that CSCs are one of the most significant obstacles in eradicating colon cancer [6–8]. Successful cancer therapy requires not only the killing of proliferating tumor cells but also the elimination of CSCs.

Napabucasin (NAP), also known as BBI608, is an orally administered CSCs inhibitor, which has been clinically evaluated to treat a variety of cancers, including pancreatic ductal adenocarcinoma, non-small-cell lung cancer, etc. [9–11]. NAP blocks the self-renewal and depleted survival of stemness-high human cancer cells, while standard chemotherapeutic drugs, such as gemcitabine or carboplatin, cause the enrichment of the subpopulation of these cells [12, 13]. Although NAP appears to target both the CSCs and non-CSCs in the bulk of the tumor, the growth and survival of normal cells appear to be unaffected [14]. NAP has perfect anti-tumor effect in vitro studies, but phase III clinical trials have not yielded positive results in vivo. This might be connected to NAP’s poor solubility in water and low bioavailability. NAP additionally causes digestive side effects [15]. Improvement in the tumor-targeting capabilities of NAP will help in the advancement of bioavailability and reduction of toxic side effects, thereby advancing its clinical applications. Drug transporters can help deliver NAP to tumors more effectively.

Tumor-derived microparticles (TMPs), derived from apoptotic tumor cells, are the ideal drug transport carriers [16–18]. TMPs are extracellular vesicles of 100–1000 nm, isolated from ultraviolet-irradiated tumor cells and formed as an outgrowth of the tumor cell plasma membrane [19, 20]. TMPs are natural nanocarriers that have several distinctive advantages. For example, as carriers, they can improve the defect of poor tumor targeting. By using their membrane surface antigens to mediate the inherent homotypic adhesion property, TMPs have the ability to specifically target tumor homologous cancer cells [21–23]. By transporting a collection of tumor antigens, co-stimulatory molecules, and DNA fragments resembling their parental cells, TMPs can also activate the immune system's anti-tumor defenses [24]. TMPs have a high level of biosafety and clinical translation potential. The progression of malignant pleural effusions in patients with lung cancer has been shown to be delayed by TMPs derived from the patients, which are currently being tested in clinical settings [20, 25].

**Scheme 1:**
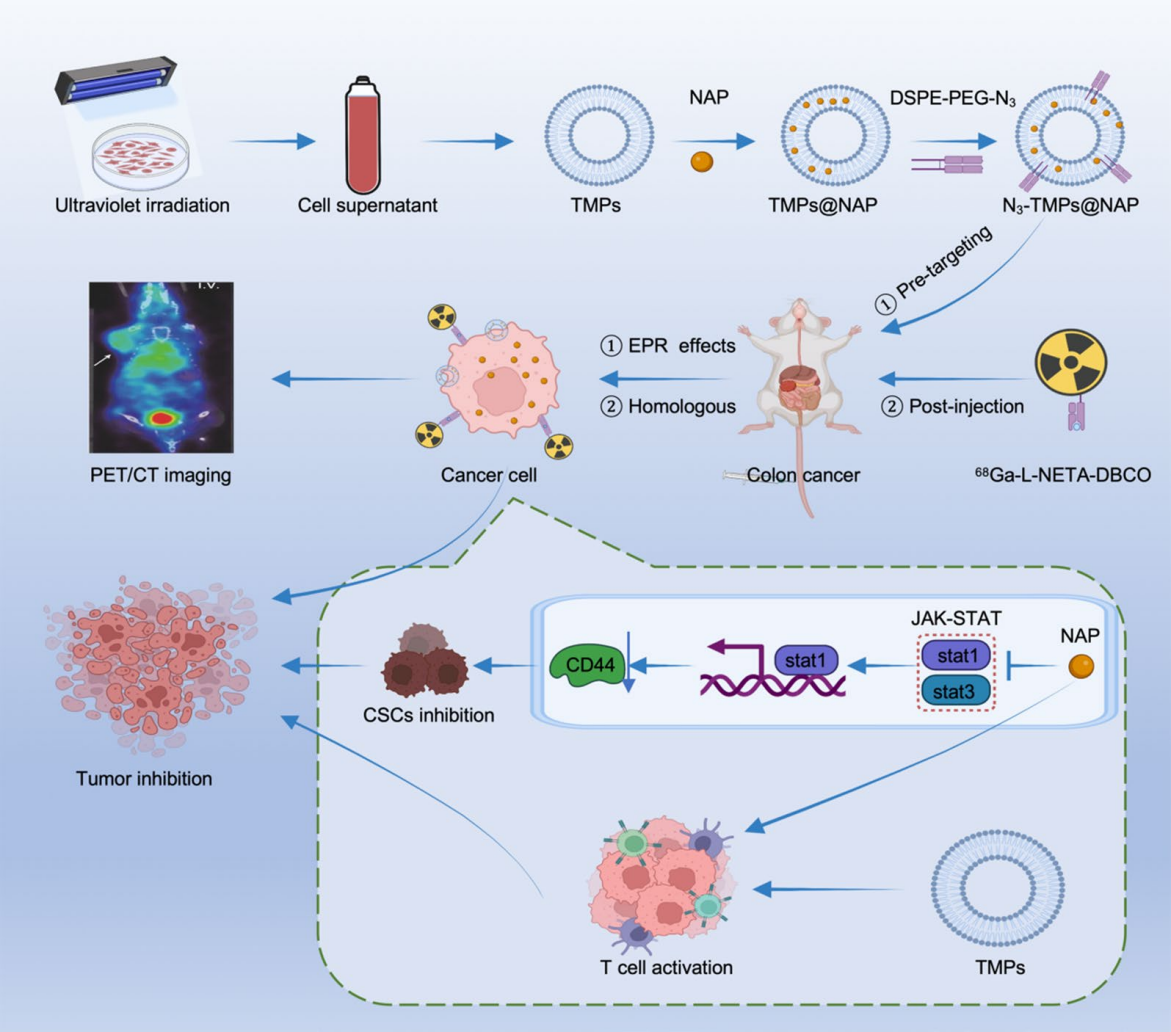
Schematic representation of synthesis, application, and analysis of TMPs-based nanoprobe

Herein, the novel mechanism of NAP inhibiting tumor was elucidated for the modification of clinical application scheme. Additionally, a TMPs-based integrated diagnosis and treatment probe was developed to increase NAP's bioavailability and synergistically activate immune responses against cancer. PET/CT imaging based on pre-targeting strategy was used to monitor cancer progression. The mechanism of tumor inhibition by the nanoprobe is studied using bioinformatics and molecular biology, providing a theoretical foundation for clinical transformation (Scheme 1).

## Results and discussion

### Bioinformatics analysis of the antitumor effect of NAP

**Fig. 1:**
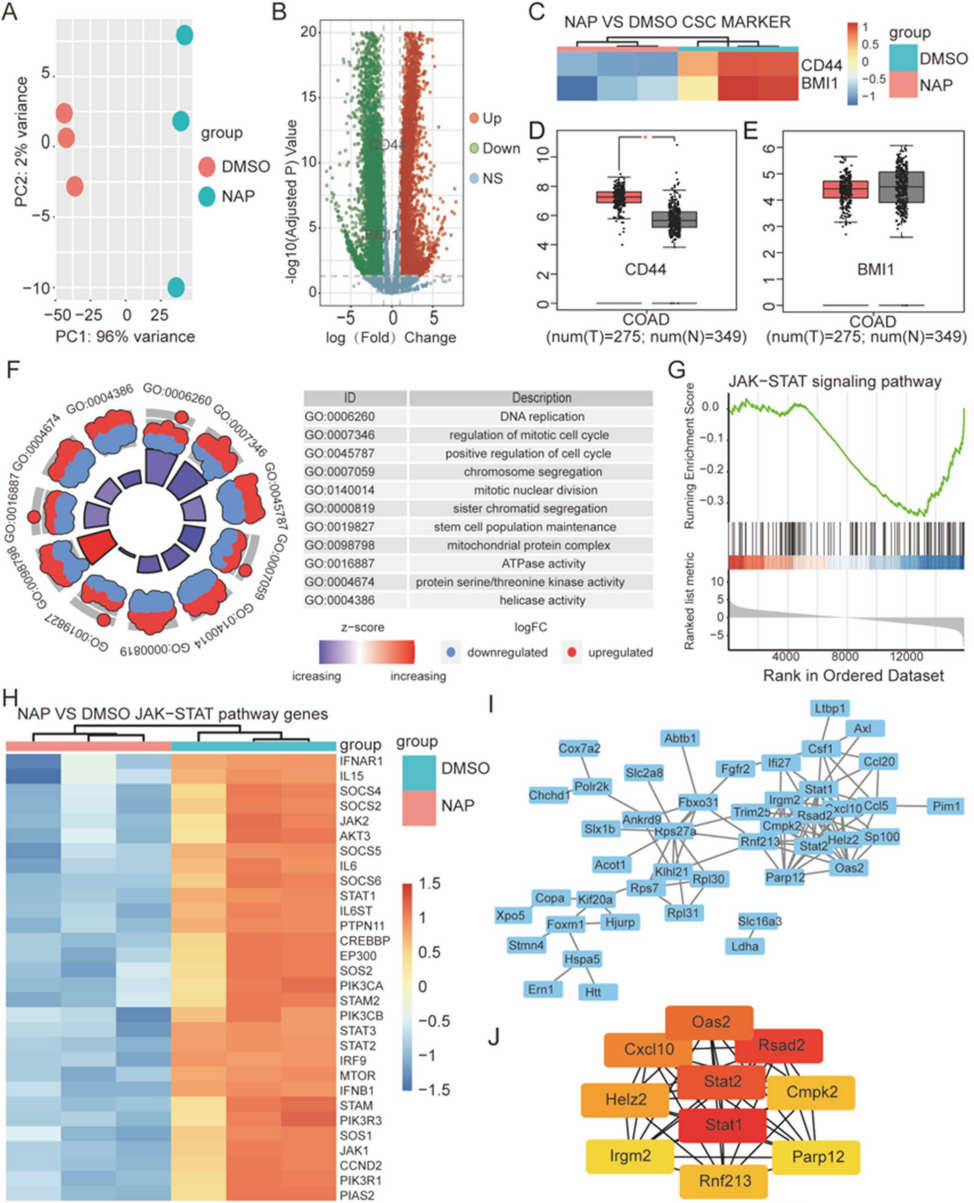
Bioinformatics analysis of the antitumor effect of NAP. **A** The analysis of the quality control of RNA sequencing. **B** The change in the number of genes after NAP treatment. **C** After the NAP treatment, the tumor stem cell markers, CD44, and BMI1 were significantly down-regulated. **D** The expression of *CD44* in colon cancer patient tissues. **E** The expression of *BMI1* in colon cancer patient tissues. **F** Changes of many biological processes after NAP treatment. **G** GSEA algorithm was used to analyze the changes in the JAK-STAT pathway after NAP treatment. **H** Significant changes in the expression levels of genes involved in the JAK-STAT pathway. **I** PPI network of 100 genes with the most significant changes. **J** The top 10 hub genes

The control and NAP-treated CT26 mouse colon cancer cells were subjected to RNA sequencing (RNA-seq) analysis to investigate the underlying mechanism of inhibiting colon cancer cells by NAP. The quality assurance of RNA-seq was examined using principal component analysis (PCA) (Fig. 1A). Figure 1B depicted the change in genes after NAP treatment. Considering that NAP could inhibit tumor cells' stemness, the intersection of different genes with tumor stem cell markers revealed that CD44 and BMI1 expression levels were noticeably down-regulated following NAP treatment (Fig. 1C). The expression of CD44 and BMI1 was then examined in colon cancer patient tissues, and it was discovered that CD44 was expressed more highly in cancerous tissues than in healthy colon tissue. While there was no statistically significant difference between the expression of BMI1 in healthy colon tissue and colon cancer (Fig. 1D, E). Some biological processes, such as controlling stem cell differentiation and maintaining stem cell population, were significantly down-regulated, according to GO enrichment analyses. These outcomes supported the earlier investigation and suggested that NAP might inhibit colon cancer stem cells. As expected, DNA replication and cell cycle were markedly suppressed by NAP treatment.

Interestingly, it was also discovered that the biological process of neutrophil activation in the immune response was upregulated after NAP treatment, indicating the triggering of immune response by NAP (Fig. 1F). After NAP treatment, the GSEA algorithm discovered that the JAK-STAT pathway was downregulated (Fig. 1G). Considering that NAP might inhibit the JAK-STAT pathway, so significant changes in the expression levels of JAK-STAT pathway genes were examined. This revealed that NAP could down-regulate a number of crucial genes, including JAK1, JAK2, STAT1, STAT2, and STAT3 (Fig. 1H). The STRING database was used to create the PPI network of the top 100 changed genes, which was then visualized using Cytoscape (Fig. 1I). The Mcode algorithm was used to determine the top 10 hub genes (Fig. 1J). Subsequently, we found that STAT1 played a crucial role in the PPI network and was the first significant node among the top 10 hub genes. These findings demonstrated that NAP could suppress the JAK-STAT pathway and inhibit cancer stemness. Notably, the bioinformatics analysis suggested that, in addition to inhibiting STAT3 as reported in the literature [10, 13], NAP might also inhibit STAT1 and exert a tumor suppressor effect.

### NAP suppresses STAT1 expression to inhibit colon cancer

**Fig. 2:**
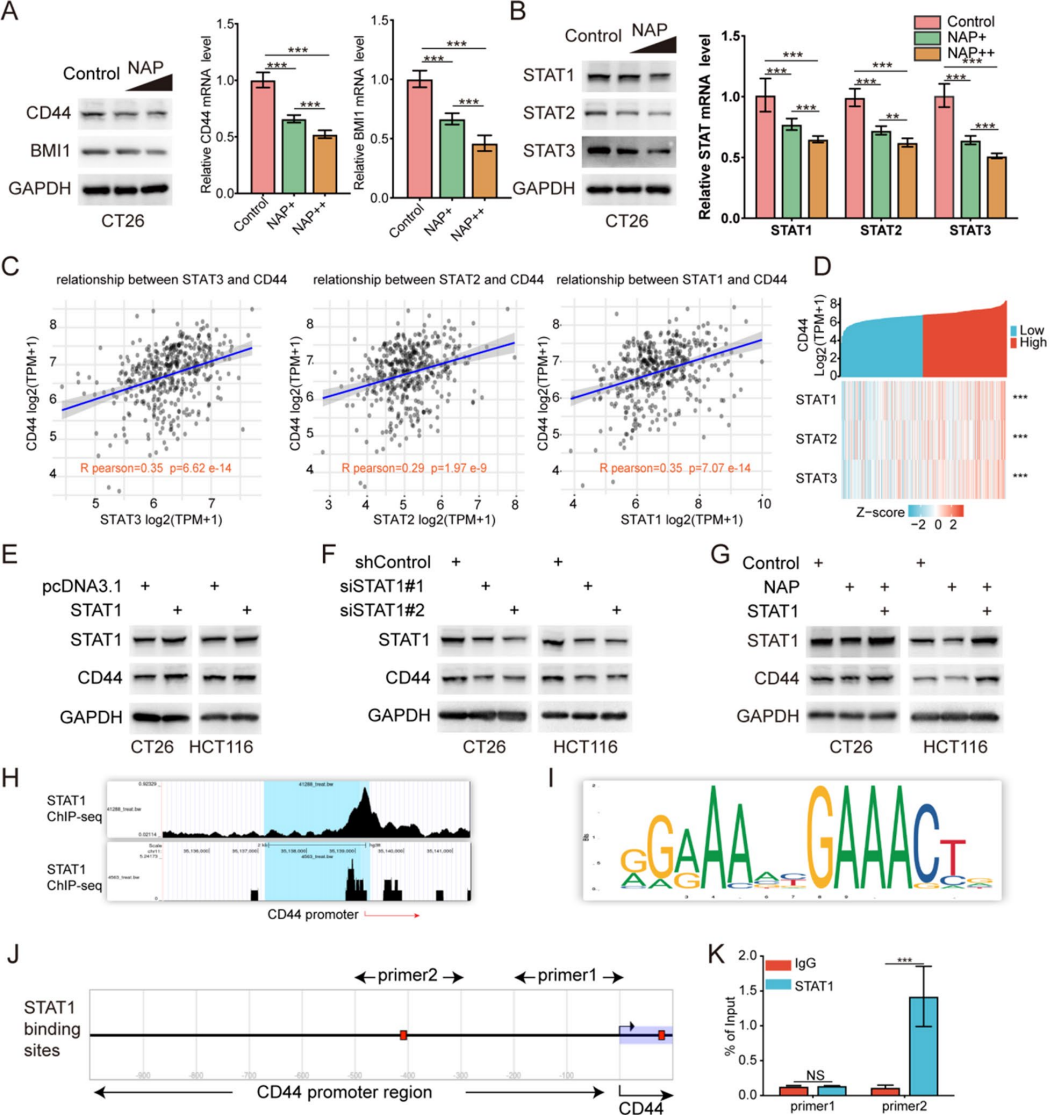
NAP inhibited colon cancer by reducing STAT1 expression. **A** Western blot and RT-qPCR analysis of CD44 and BMI1 in NAP-treated CT26 cells. **B** Western Blot and RT-qPCR analyses of STAT1, STAT2, and STAT3 in the NAP-treated CT26 cells. **C** The correlations of STAT1, STAT2, and STAT3 with CD44 in human colon cancer tissues. **D** The heatmap of STAT1, STAT2, and STAT3 with CD44 in human colon cancer tissues. **E** Overexpression of STAT1 increased the protein levels of CD44. **F** Knockdown of STAT1 decreased the protein levels of CD44. **G** The overexpression of STAT1 reversed the effects of NAP on CD44 inhibition. **H** A STAT1 binding peak in the promoter region of CD44. **I** A consensus binding motif for STAT1. **J** The presence of STAT1 binding sites. **K** STAT1 bound to the promoter region of CD44

To further verify that NAP could inhibit colon cancer stemness, Western blot and RT-qPCR analyses were used to detect the protein and mRNA expression levels of CD44 and BMI1 in the CT26 cells. The CT26 cells were treated with various concentrations of NAP (0.5 μM, 1 μM). The results showed that levels of protein and mRNA expression decreased as NAP concentration increased (Fig. 2A). To evaluate the inhibitory effect of NAP on STAT1, STAT2, and STAT3, Western Blot and RT-qPCR analyses were used to evaluate the changes in the protein and mRNA expression levels after NAP treatment (0.5 μM and 1 μM NAP). As expected, NAP could significantly inhibit the protein and mRNA expression levels of STAT1, STAT2, and STAT3 (Fig. 2B). According to some reports, STAT3 regulates the expression of CD44, and NAP can inhibit tumor stemness by inhibiting STAT3 [26] [27]. STAT1 plays a central role in PPI network analysis after NAP treatment. As a result, we investigated how NAP affected STAT1 to down-regulate CD44. First, STAT1, STAT2, and STAT3 correlations with CD44 in human colon cancer tissues were examined. TCGA-COAD data showed that the mRNA expression levels STAT1, STAT2, and STAT3 were significantly positively correlated with CD44, and the R Pearson between STAT1 and CD44 was 0.35, which was the same as that between STAT3 and CD44 (Fig. 2C). This suggested that STAT1 might transcriptionally regulate CD44 similar to STAT3. Similarly, the heatmap showed that the expression level of STAT1 significantly increased in colon cancer patients with high CD44 expression (Fig. 2D). To further prove that STAT1 regulated the expression of CD44, STAT1 was overexpressed/knockdown in the CT26 and HCT116 cells. The results showed that the STAT1 overexpression increased the protein levels of CD44 (Fig. 2E), while its knockdown decreased the protein levels of CD44 in the colon cancer cells (Fig. 2F). The effects of NAP (0.5 μM) on CD44 inhibition were reversed by the overexpression of STAT1 (Fig. 2G). These results suggested that STAT1 upregulated CD44 expression, while NAP might reduce the expression of CD44 by down-regulating the STAT1. As a transcription factor, STAT1 might promote the expression of CD44 similar to STAT3, thereby promoting the stemness of colon cancer cells. To further confirm this speculation, the published ChIP-seq data (GSM1057012, GSM671396) were analyzed, which consistently showed that there was a binding peak of STAT1 in the promoter region of CD44 (Fig. 2H) [28, 29]. Then, the JASPAR database (http://jaspar.genereg.net) was used to analyze the promoter region of CD44, revealing a consensus binding motif for STAT1 (Fig. 2I). In addition, the CD44 promoter analysis using EPD (https://epd.epfl.ch//index.php) showed the presence of STAT1 binding sites (Fig. 2J). The subsequent ChIP-qPCR indicated that STAT1 bound to the promoter region of CD44 (Fig. 2K). These results indicated that NAP inhibits CD44 by lowering STAT1 expression. STAT1 could transcriptionally regulate the expression levels of CD44.

### Preparation and characterization of TMPs and N_***3***_***-TMPs@NAP***

**Fig. 3:**
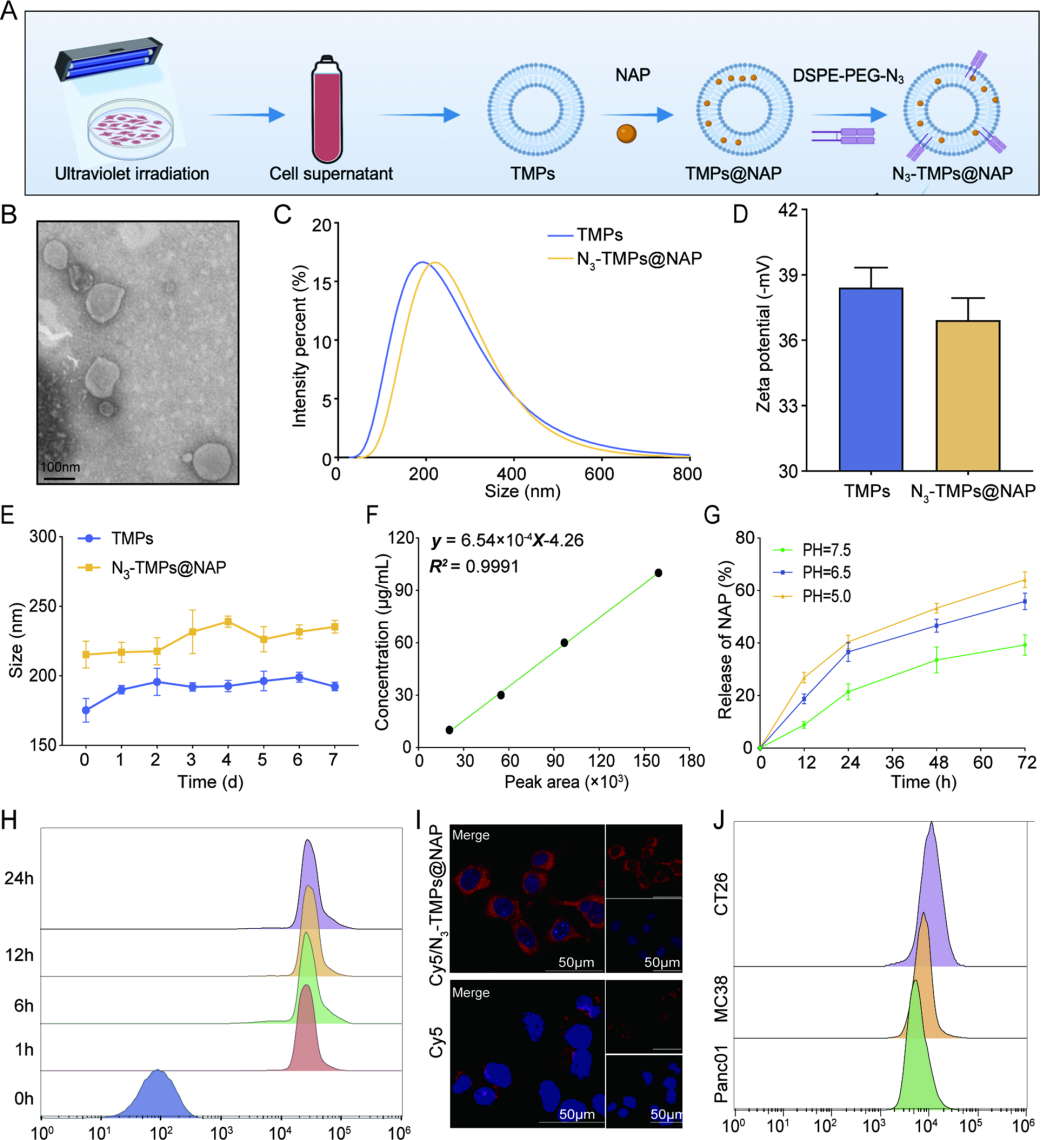
Characterizations of N_3_-TMPs@NAP. **A** Schematic representation of N_3_-TMPs@NAP synthesis. **B** TEM images of N_3_-TMPs@NAP. Scale bars = 100 μm. **C** The average hydrodynamic diameters of TMPs and N_3_-TMPs@NAP. **D** Zeta potentials of TMPs and N_3_-TMPs@NAP. **E** The hydrodynamic diameters of TMPs and N_3_-TMPs@NAP in PBS over 7 days. **F** The standard curve of NAP. **G** In vitro release of NAP from N_3_-TMPs@NAP at different PH. **H** Flow cytometric analysis of CT26 cells treated with Cy5/N_3_-TMPs@NAP or free cy5. **I** Fluorescence images of CT26 cells treated with Cy5/N_3_-TMPs@NAP. **J** Flow cytometric analysis of CT26 cells, MC38 cells and Panc01 cells treated with Cy5/N_3_-TMPs@NAP

This study selected CT26 cells as the donor cells for TMPs production. The protocol was based on previous studies and presented in Fig. 3A [30]. Using transmission electron microscope (TEM), the TMPs showed an irregular spherical morphology (Fig. 3B). Dynamic light scattering (DLS) analysis revealed that the isolated TMPs had a mean diameter of 180.50 ± 2.38 nm, while the diameter of N_3_-TMPs@NAP ranged from 40 to 970 nm with a mean diameter of 220.13 ± 4.52 nm (Fig. 3C). The nanoparticle tracking analysis (NTA) showed the particle sizes of TMPs and N_3_-TMPs@NAP (Additional file 1: Fig. S1). The zeta potentials of TMPs and N_3_-TMPs@NAP were − 38.40 ± 0.93 mV and − 36.92 ± 1.01 mV, respectively (Fig. 3D). The average hydrodynamic diameters of TMPs and N_3_-TMPs@NAP did not change significantly for up to 7 days, indicating their excellent stability (Fig. 3E). The standard curve of NAP is shown in Fig. 3F. The amount of NAP loaded into TMPs was analyzed using high-performance liquid chromatography (HPLC). The efficiency of NAP for encapsulating N_3_-TMPs@NAP was 31.17 ± 2.50%. The release profile of NAP was studied in phosphate buffered saline (PBS) at 37 °C, which showed that 39.27 ± 3.87% of the NAP was released from the N_3_-TMPs@NAP after 72 h of incubation, indicating the good stability of TMPs loading (Fig. 3G). The PH of tumor microenvironment is 6.5–7.0. The release of the agent under acidic conditions has been added, up to 64.17 ± 3.03% when PH was 5.0. Our experiments also prove that TMPs can release more drugs at tumor sites. As shown in flow cytometric analysis and fluorescence images, N_3_-TMPs@NAP had good tumor-targeting ability with CT26 cells ( Fig. 3H, I). In addition, Cy5/N_3_-TMPs@NAP were incubated with 3 cancer cell lines before being detected by flow cytometry. The outcomes showed that CT26 cells had the strongest fluorescence signal and that TMPs are capable of homologous targeting (Fig. 3J). These data showed that TMPs were ideal nanocarriers, and N_3_-TMPs@NAP was successfully constructed.

### In vitro* evaluation of anti-tumor effect*

**Fig. 4:**
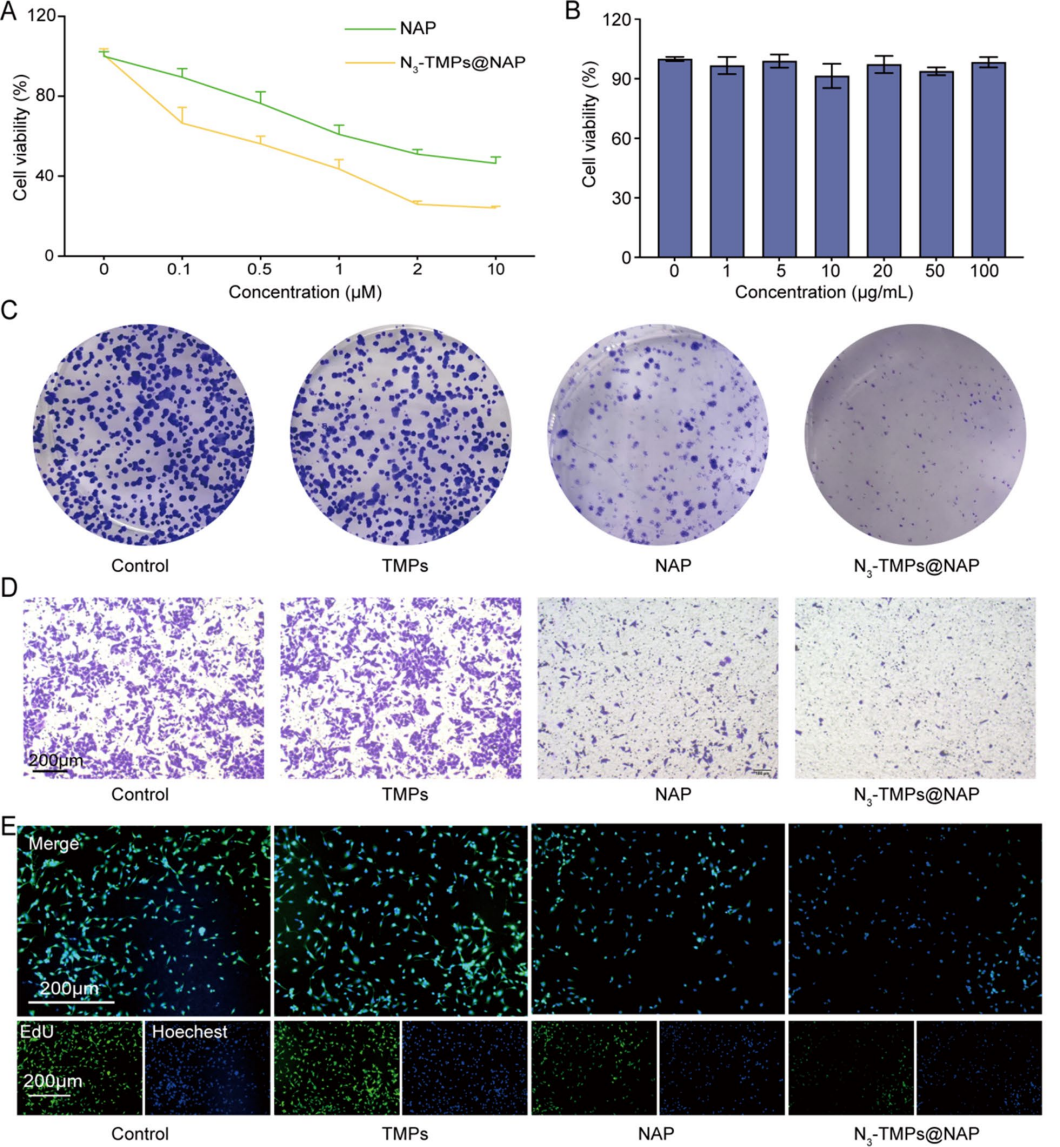
In vitro antitumor effect and in vivo toxicity. **A** In vitro cell viabilities of CT26 cells incubated with NAP and N_3_-TMPs@NAP. **B** In vitro cell viabilities of CT26 cells incubated with TMPs. **C** In vitro anti-tumor effect detected by colony formation assay. **D** In vitro anti-tumor effect detected by transwell invasion assay. **E** In vitro anti-tumor effect detected by EdU assay. Scale bars = 200 μm. Data are represented as mean ± SD (n = 3)

CCK-8 assay was performed to evaluate the anti-tumor effect of NAP and N_3_-TMPs@NAP in the CT26 colon cancer cells. The CT26 cells were co-incubated with NAP and N_3_-TMPs@NAP at different concentrations of NAP (up to 10 μM). With increasing concentrations, the survival rate of cells in the N_3_-TMPs@NAP group decreased, and fell below that of the NAP group's cells (Fig. 4A). Additionally, TMPs were co-incubated with CT26 cells at a range of concentrations (up to 100 μg/mL). The results showed that each group's cell survival rate was higher than 90%, indicating that the TMPs had minimal in vitro cytotoxicity (Fig. 4B). CT26 cells in the N_3_-TMPs@NAP group exhibited the lowest proliferative and invasive abilities, as determined via a colony formation assay and transwell assay (Fig. 4C, D). The N_3_-TMPs@NAP group's lowest fluorescence signals were observed in EdU assay, indicating the best anti-tumor effect (Fig. 4E). Live and dead cell staining experiments of CT26 cells were displayed in Additional file 1: Fig. S2. TMPs had no antitumor effect in vitro as evidenced by the fact that the fluorescence signals of the TMPs group were comparable to those of the control group. However, TMPs were able to substantially raise the level of NAP in tumor cells, leading to a fantastic anti-tumor effect N_3_-TMPs@NAP.

### In vivo* PET/CT imaging and biodistribution analysis*

**Fig. 5:**
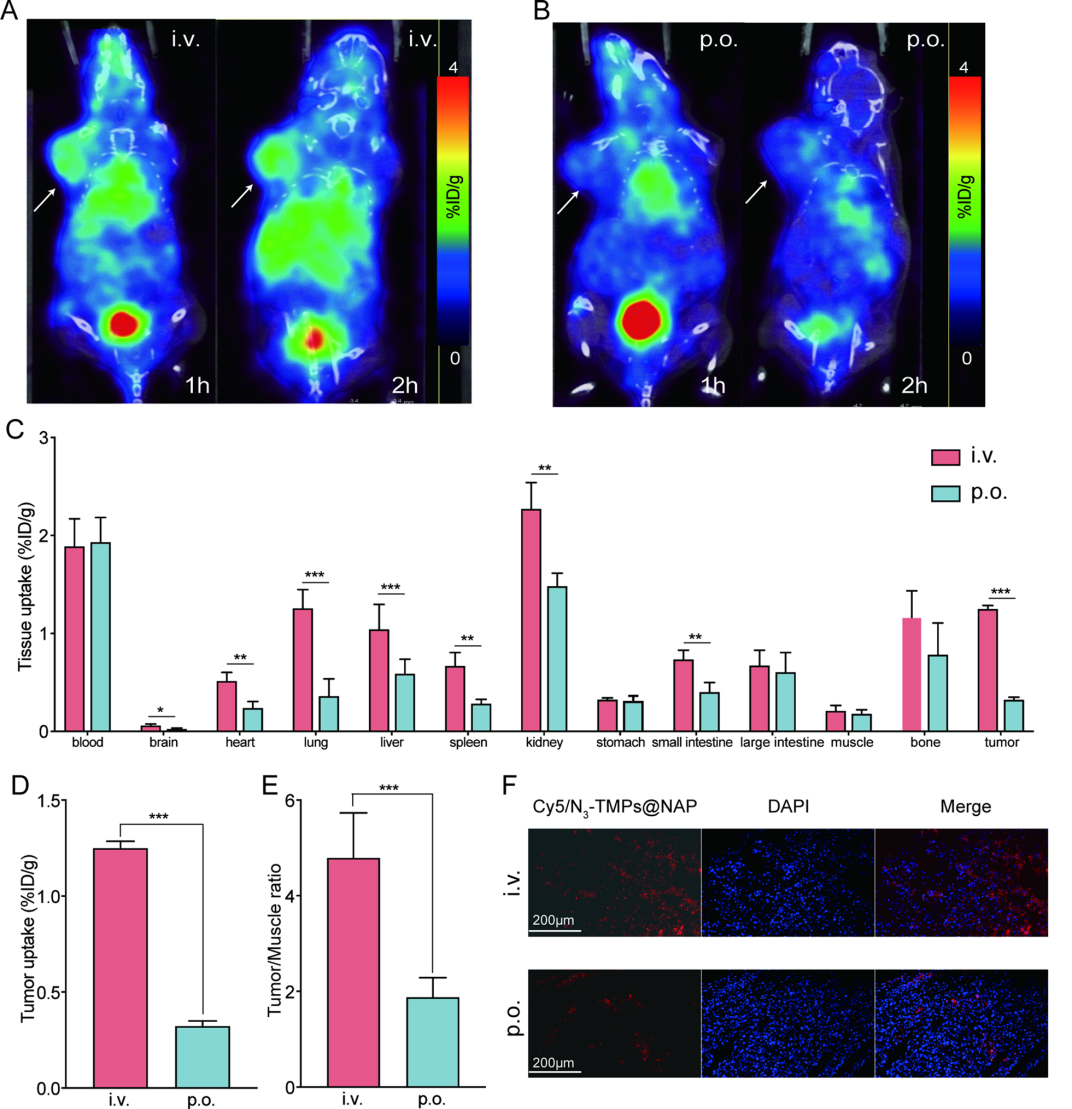
In vivo PET/CT imaging and biodistribution of CT26 tumor-bearing mice. **A** PET/CT images of CT26 tumor-bearing mice after tail vein injection of N_3_-TMPs@NAP (i.v.) for 20 h, and intravenous injection of ^68^ Ga-L-NETA-DBCO for 1 h, 2 h. **B** PET/CT images of CT26 tumor-bearing mice after oral administration of N_3_-TMPs@NAP (p.o.) for 20 h, and intravenous injection of ^68^ Ga-L-NETA-DBCO for 1 h, 2 h. **C** Tissues uptakes of CT26 tumor-bearing mice at 2 h after the injection of ^68^ Ga-L-NETA-DBCO. **D**–**E** Tumor uptake and tumor to muscle uptake ratio at 2 h after the injection of.^68^ Ga-L-NETA-DBCO. **F** Fluorescence images of tumor tissues after i.v. or p.o. of Cy5/N_3_-TMPs@NAP. Scale bars = 200 μm. Data are represented as mean ± SD (n = 3; *P < 0.05; **P < 0.01; ***P < 0.001)

According to Additional file 1: Fig. S3, ^68^ Ga-L-NETA-DBCO may be helpful for PET/CT imaging of tumors. However, when combined with a click reaction with N_3_-TMPs@NAP, the tumor uptakes were higher than those of ^68^ Ga-L-NETA-DBCO. Additionally, as more N_3_-TMPs@NAP was injected into the mice, the tumor uptakes increased. Previous study discovered that the best imaging period for PET/CT imaging was 2 h and the best pre-targeting period was 20 h [30]. The maximum tumor uptakes by tumor tissues were observed in the tail intravenous group at 2 h following the injection of ^68^ Ga-L-NETA-DBCO when comparing the two different delivery methods of N_3_-TMPs@NAP (oral group and tail intravenous group) (Additional file 1: Figs. S4, S5A, B). According to the biodistribution results, the uptake of tumor tissues in the tail intravenous group was higher than that of tumor tissues in the oral group. Moreover, the uptakes by the tissues of the heart, lung, kidney, liver, and spleen tissues in the tail intravenous group model were also higher than those in the oral group (Figs. 5C, D). Furthermore, the tail intravenous group's tumor to muscle ratio and Cy5/N_3_-TMPs@NAP fluorescence signals were both higher than those in the oral group (Figs. 5E, F). As a result, the N_3_-TMPs@NAP showed good bioavailability when injected into the tail vein and accumulated more in the tumor tissues.

### In vivo* antitumor effect assessment*

**Fig. 6:**
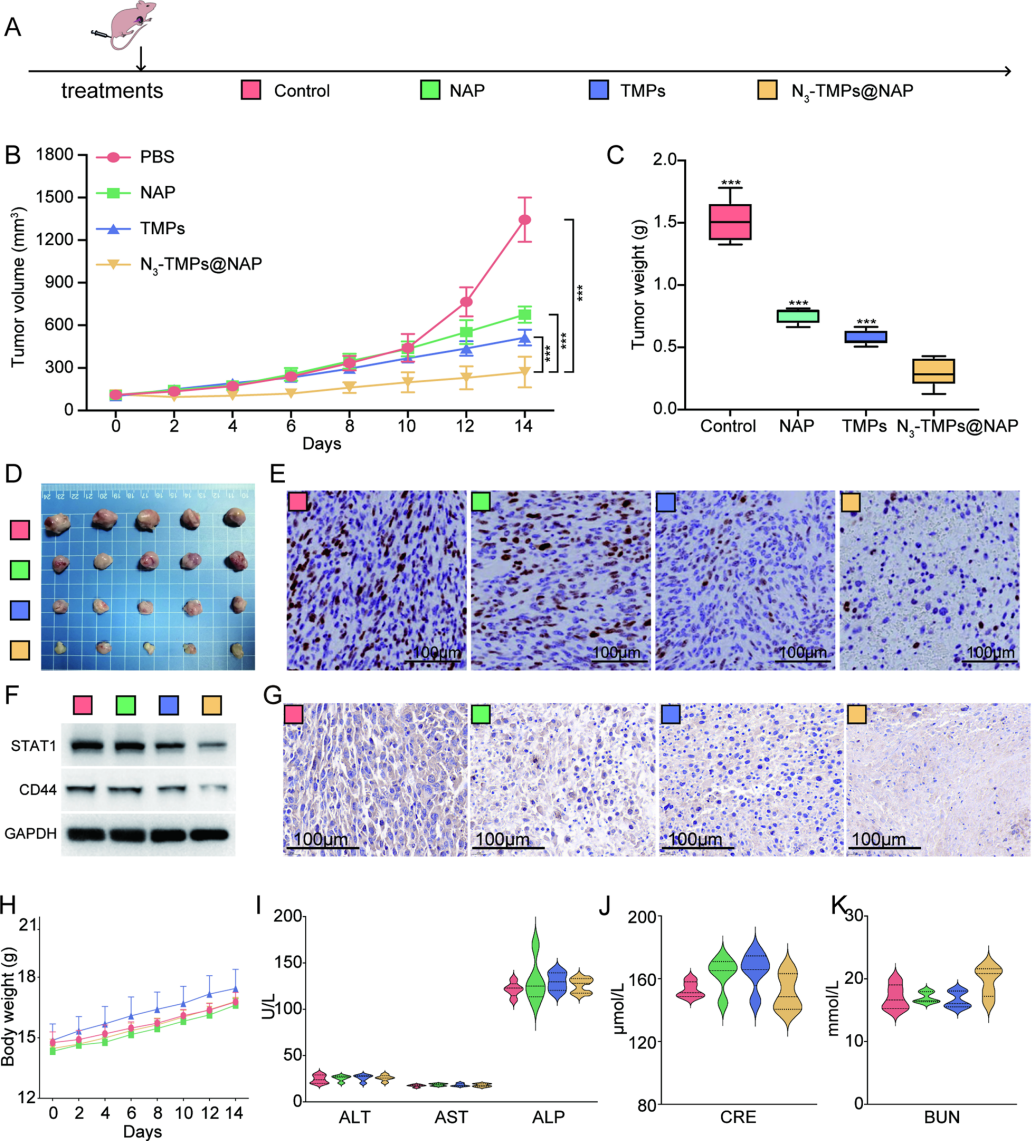
In vivo antitumor effect and in vivo toxicity. **A** The therapeutic schedule. **B** Tumor growth curves in each group at given time points. **C** Average tumor weights in each group in the end. **D** Representative images of CT26 tumor tissues in the end. **E** Immunohistochemical staining of Ki67 in tumor tissues. **F** Western blot analysis of CD44 and STAT1 in CT26 tumor-bearing mice after treatment. **G** Immunohistochemical staining of CD44 in CT26 tumor tissues. **H** Body weight change curves. **I**–**K** Liver function markers (ALT, AST, and ALP) and kidney function markers (BUN and CRE) after different treatments

The treatment schedule and grouping of tumor-bearing mice are shown in Fig. 6A. After treatment, the tumor size of the tumor-bearing mice was observed. According to the tumor growth curves (Fig. 6B), the average tumor volume in the NS group increased steadily, reaching 1345.24 ± 157.10 mm^3^ on the 14th day. The average tumor volumes in the NAP, TMPs, and N_3_-TMPs@NAP groups significantly decreased, and the N_3_-TMPs@NAP group had the smallest average tumor volume of 270.55 ± 107.59 mm^3^ on the 14th day. Additionally, the N_3_-TMPs@NAP group's average tumor weight was the lowest (0.30 ± 0.12 g), demonstrating the best treatment outcomes (Fig. 6C). Similar results were also observed in the representative images of tumor tissues (Fig. 6D). Ki67 immunohistochemical staining was used to evaluate the proliferation of tumor tissues (Fig. 6E). The levels of Ki67 staining in the tumor tissues of the N_3_-TMPs@NAP group were diminished. The data demonstrated that the N_3_-TMPs@NAP possessed effective in vivo anti-tumor properties. After treatment, the tumor tissues were gathered for Western blot analysis and immunohistochemical staining (Fig. 6F, G). The results showed a lower expression of STAT1 and CD44 in the tumor tissues of the NAP and N_3_-TMPs@NAP groups. The N_3_-TMPs@NAP group’s tumor tissues had the lowest STAT1 and CD44 protein expression levels. The N_3_-TMPs@NAP group's tumor tissues had lower levels of CD44 staining, and CD44 immunohistochemical staining was consistent with the Western blot. The *in-vivo* pharmacokinetic parameters of NAP and N_3_-TMPs@NAP were detected by HPLC (Additional file 1: Fig. S5). Two hours after tail vein injection, the concentration of NAP in serum of the N_3_-TMPs@NAP group was higher than that of NAP group of, the content of NAP in serum of TMPs group was higher than that of NAP group. According to the results, N_3_-TMPs@NAP could prolong the blood concentration of NAP and increase the bioavailability of NAP,

### In vivo* toxicity studies*

There was no obvious difference in the weights of the mice across all groups, indicating that the treatments were not significantly toxic (Fig. 6H). The liver and kidney function indicators, including alanine amino transferase (ALT), aspartate aminotransferase (AST), alkaline phosphatase (ALP), blood urea nitrogen (BUN), and creatinine (CRE), showed no significant hepatic or renal toxicity (Fig. 6I, K). Additionally, the hematoxylin and eosin (H&E) staining also showed no significant evidence of major organ damage (Additional file 1: Fig. S6). The results showed that the N_3_-TMPs@NAP had no significant toxicity to the normal tissues.

### In vivo* evaluation of anti-metastatic liver tumor effect*

**Fig. 7:**
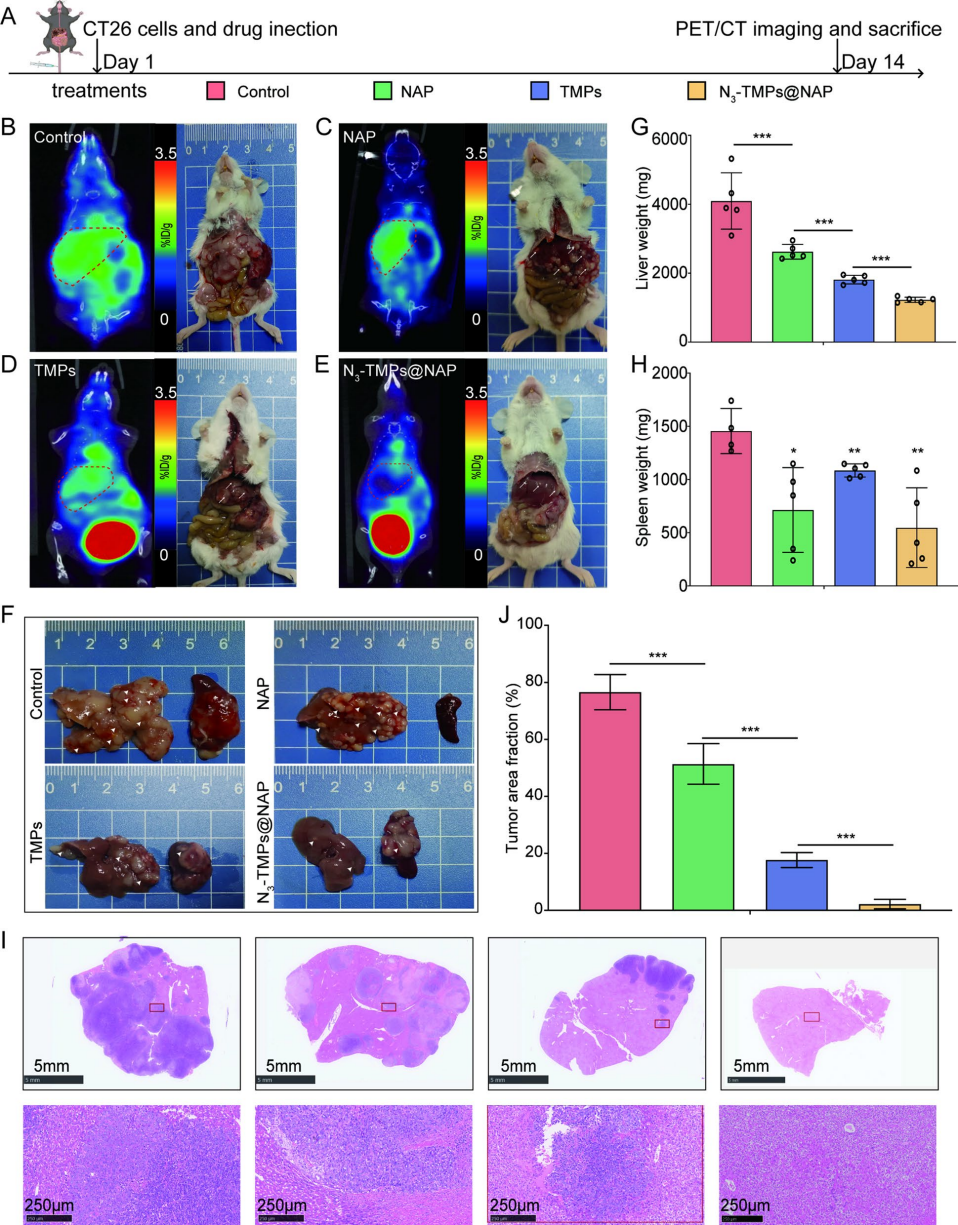
In vivo evaluation of anti-metastatic liver tumor effect. **A** The therapeutic schedule. **B**–**E** PET/CT imaging and representative mice in the end. **F** Representative liver and spleen tissues in the end. **G** The average weight of liver tissues in the end. **H** The average weight of spleen tissues in the end. **I** Representative H&E staining images of liver tissues from the euthanized mice. **J** The tumor area fraction of liver tissues

Figure 7A illustrated the treatment plan and classification of the colon cancer liver metastases models. PET/CT imaging was carried out after 14 days using the selected optimal imaging settings. PET/CT images show the morphology and radioactive uptake of the livers in each group (Fig. 7B–E). The morphology and radioactive uptake of the livers in each group can be observed on PET/CT images (Fig. 7B–E). As expected, the fewest liver metastases were detected in the N_3_-TMPs@NAP group. Similar results were observed in the representative liver metastasis images (Fig. 7F). The average liver weight in the N_3_-TMPs@NAP group was the lowest (Fig. 7G) and the average spleen weight was the highest in the control group (Fig. 7H). The H&E staining and corresponding quantitative analysis also showed the smallest area of liver metastases in the N_3_-TMPs@NAP group (Fig. 7I, J). These findings further showed that N_3_-TMPs@NAP had potent anti-tumor properties and could prevent colon cancer liver metastases.

### In vivo* immune response*

**Fig. 8:**
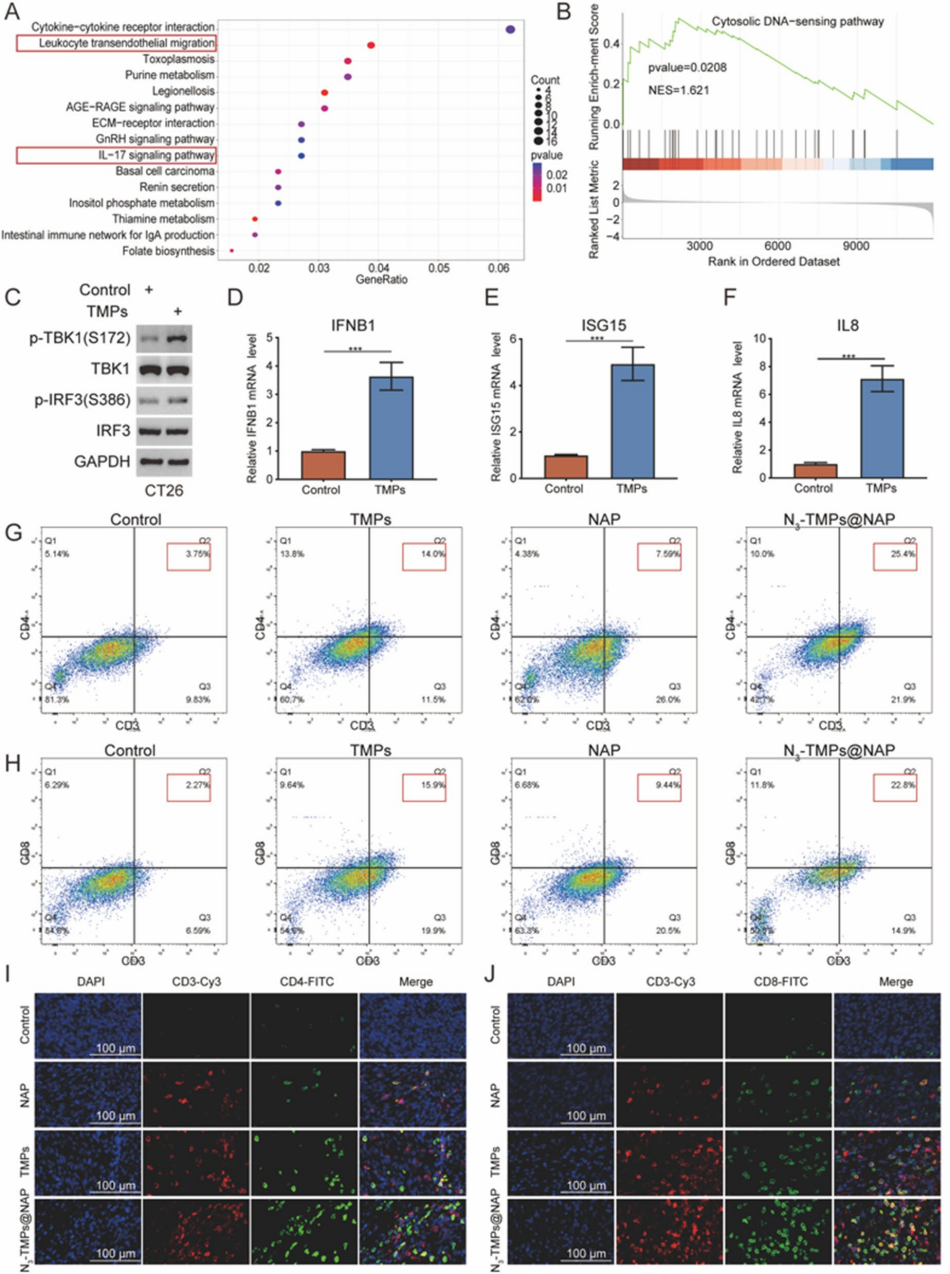
In vivo validation of the mechanism of NAP inhibiting colon cancer. **A** The KEGG enrichment analysis indicated that the immune-related pathway was regulated after TMPs treatment. **B** GSEA algorithm was used to analyze the changes in the cytosolic DNA-sensing pathway after TMPs treatment. **C** p-TBK1(Ser172) and p-IRF3(Ser386), markers of activation of innate immune pathway cytosolic DNA-sensing, were detected after TMPs treatment. **D**–**F** RT-qPCR detected downstream target genes of the DNA-sensing pathway. **G**–**H** Representative flow analysis of CD3^+^CD4^+^ T cells and CD3^+^CD8^+^ T cells in tumor tissues. **I**–**J** Representative fluorescent images of CD3^+^CD4^+^ T cells and CD3^+^CD8^+^ T cells in tumor tissues. (n = 6, scale bars:100 μm)

To further explore the underlying mechanism by which TMPs inhibit colon cancer cells, the control, and TMPs-treated CT26 cells were subjected to RNA-seq analysis. KEGG enrichment analysis indicated that the immune-related signaling pathway was regulated after TMPs treatment, including leukocyte transendothelial migration and the IL-17 signaling pathway (Fig. 8A). The GSEA algorithm was used to examine the changes in the immune-related pathway following TMPs treatment in order to confirm whether or not TMPs could stimulate an immune response (Fig. 8B). After CT26 cells were treated with TMPs (50 μg/mL), the markers of innate immune cGAS-STING pathway activation, including p-TBK1(Ser172) and p-IRF3(Ser386), were significantly increased (Fig. 8C). In addition, RT-qPCR demonstrated that the downstream ISG15, IFNB1, and IL8 of the innate immune pathway were also considerably increased after TMPs treatment (Fig. 8D–F). The results indicated that TMPs could promote the innate immune pathway of tumor cells, which may be related to the double-stranded DNA encapsulated by TMPs [31–33]. As expected, the cytosolic DNA-sensing pathway was upregulated. Then the immunomodulatory effects of each group were evaluated based on the subpopulations of CD3^+^CD4^+^ helper and CD3^+^CD8^+^ cytotoxic T cells using the flow analysis and immunofluorescence assay of tissues (Fig. 8G–J). The number of CD3^+^CD4^+^ and CD3^+^CD8^+^ T cells significantly increased in the NAP, TMPs, and N_3_-TMPs@NAP groups, with the N_3_-TMPs@NAP group showing the highest growth of the T cell subpopulation, indicating a potent antitumor immune response.

## Conclusions

In this study, an integrated nanoprobe was prepared to diagnose and treat colon cancers using the TMPs as a nanocarrier. The nanoprobe was used successfully to track the development of colon cancer using PET/CT imaging based on a pre-targeting strategy. By suppressing CSCs and inducing an immune response, the nanoprobe may be able to prevent colon cancer by utilizing NAP's functionality. The mechanism of NAP inhibiting CSCs was further explored, showing that STAT1 could bind to the CD44 promoter to regulate its expression, and NAP could inhibit CD44 expression via suppressing the STAT1. In addition, the nanoprobe could activate the anti-tumor immune response more effectively with the application of TMPs. However, we did not conduct a clinical study in this study. In the following clinical research, we will adopt TMPs from human tumor tissue (obtained by surgery or puncture) or tumor cells (obtained from pleural effusion or ascites). Overall, the strategy described herein is versatile and is not only confined to colon cancer but holds great promise for treating a variety of tumors in clinics.

## Methods

### Cell culture

HCT116 human colon cancer cell, CT26 mouse colon cancer, MC38 mouse colon cancer cell, and human pancreatic cancer cell were preserved in our laboratory (Hubei Province Key Laboratory of Molecular Imaging, China). The cells were cultured in an RPMI-1640 medium (Gibco, USA) supplemented with 10% fetal bovine serum (FBS, Gibco, USA) and incubated at 37 °C in a humidified atmosphere with a 5% CO_2_ concentration.

### Isolation of TMPs

The CT26 cells were cultured in RPMI-1640 medium supplemented with 10% FBS (without extracellular vesicles) after being exposed to ultraviolet irradiation (300 J/m^2^) for 1 h. The supernatants were used to isolate TMPs after 24 h. Briefly, the supernatants were centrifuged for 60 min at 14,000 g to pellet the TMPs after being centrifuged for 30 min at 3000 g to remove cells. The TMPs pellets were resuspended in a culture medium after being cleaned three times. The TMPs were put through a series of 0.45-m filters, quantified by surface proteins using a BCA Protein Assay Kit (Beyotime, Shanghai, China), and then stored at 80 °C.

### Synthesis of N_3_-TMPs@NAP

The protocol was referred to previous study [34]. Dimethylsulfoxide (DMSO, Solarbio, China) was used as a permeability enhancer by increasing the solubility of NAP and its permeability across the lipid membrane of TMPs. A 4% (v/v) DMSO solution in PBS was used for the NAP encapsulation. TMPs@NAP was fabricated by mixing the TMPs with NAP and incubating them for 12 h. Then 1,2-distearoyl-sn-glycero-3-phosphoethanolamine-N-[azido (polyethyleneglycol)-2000] (DSPE-PEG-N_3_, 1 μM) was incubated with TMPs@NAP (TMPs, 1 mg) for 30 min at 37 °C to form N_3_-TMPs@NAP. The samples were then passed through centrifugal filter devices (100 kDa molecular weight, Amicon^®^Ultra-15) before further use.

### ***Characterization of N***_***3***_***-TMPs@NAP***

N_3_-TMPs@NAP was examined using TEM (Hitachi, Japan), DLS (Malvern Instruments Ltd., Worcestershire, UK) and NTA (PARTICLE METRIX, German). To test the in vitro stability of N_3_-TMPs@NAP, the hydrodynamic diameters were monitored for 7 days using DLS. The amount of NAP in N_3_-TMPs@NAP was measured using HPLC. Briefly, NAP was dissolved in DMSO and subsequently diluted to different concentrations with PBS (0, 0.05, 0.1, 0.2, 0.3, and 0.5 μM). The UV peaks, which corresponded to the different concentrations, were measured using HPLC, and the standard curves of NAP were plotted. N_3_-TMPs@NAP was placed in a dialysis bag (MW: 12,000 Da) and incubated with solution of different PH (7.0, 6.5, 5.0) to quantitatively determine the release profile of NAP, which was detected at 0, 24, 48, and 72 h.

### Synthesis of ^68^ Ga‑L‑NETA‑DBCO

The protocol was referred to previous study [30, 35]. By using HCl (0.05 M) as the eluent and a ^68^Ge/^68^ Ga generator, ^68^GaCl_3_ was produced. To adjust the pH of a 500-μL ^68^GaCl_3_ (187 MBq) solution to 3.7, sodium acetate was added. L-NETA-DBCO (5 nmol) was used to chelate the radionuclide ^68^ Ga for 10 min at 100 °C. After cooling the mixture, a C18 column was used to purify ^68^ Ga-L-NETA-DBCO. PET/CT imaging was used to detect the in vivo click reaction that occurred during the conjugation of ^68^ Ga-L-NETA-DBCO with N_3_-TMPs@NAP.

### In vitro* tumor cell binding*

The ability of *in-vitro* tumor cell binding was detected using flow cytometric analysis and fluorescence imaging. N_3_-TMPs@NAP were incubated with Cy5-NHS for 30 min at 37 °C to form Cy5/N_3_-TMPs@NAP. Cy5/N_3_-TMPs@NAP (30 μg/mL) were incubated with CT26 cells at 37℃ for different time points (0 h, 1 h, 6 h, 12 h, 24 h). Flow cytometric was used to detect the fluorescence signals. Cy5/N_3_-TMPs@NAP (30 μg/mL) or Cy5 (30 μg/mL) were then incubated with CT26 cells at 37 ℃ for 24 h. The cell nuclei were counterstained with 4′,6-diamidino-2-phenylindole (DAPI). The cells were fixed with paraformaldehyde and observed using a fluorescence microscope (Olympus, Japan). In addition, CT26 cells, MC38 mouse colon cancer cells, and Panc01 human pancreatic cancer cells were incubated with Cy5/N_3_-TMPs@NAP (30 μg/mL) at 37 °C for 24 h to further studying the capability of homologous targeting of TMPs.

### Cell counting kit-8 (CCK-8) assays and live/dead cell staining

CT26 cells were plated in 96-well plates (4000 cells/well) in triplicates and incubated at 37 °C overnight. The cells were subsequently treated with NAP (0.1, 0.5, 1, 2, and 10 μM), vehicle control (0.1% DMSO), or N_3_-TMPs@NAP (NAP: 0.1, 0.5, 1, 2, and 10 μM) for 24 h. Finally, the cell growth was measured using CCK-8 (Dojindo, Japan), following the manufacturer’s instructions. Briefly, after 1-h incubation with CCK-8 at 37 °C, their OD values (at 450 nm) were detected to calculate cell viability. TMPs were also incubated with CT26 cells at different concentrations (0, 1, 5, 10, 20, 50, and 100 μg/mL) for 24 h. To further detected the *in-vitro* anti-tumor effect, we used the LIVE/DEAD cell double staining kit (BJBALB, China) according to the manufacturer's instructions.

### Colony formation assay

The colony formation assay was used to assess tumor cell proliferation. CT26 colon cancer cells were plated into 6-well plates (500 cells per well) and treated with control (0.1% DMSO), NAP (0.05 μM), TMPs (1 μg/mL), or N_3_-TMPs@NAP (NAP 0.05 μM). After 12 days, the cells were fixed in methanol for 15 min, stained with 1% Crystal Violet Staining Solution for another 20 min, and washed 3 times with PBS. The number of colonies was counted. All assays were performed in triplicates.

### Cell invasion assay

Utilizing transwell chambers with 8-μm pore size, cell invasion capacity was evaluated. CT26 cells were treated with control (0.1% DMSO), NAP (0.1 μM), TMPs (1 μg/mL), or N_3_-TMPs@NAP (NAP 0.1 μM) and injected into the Matrigel-coated invasion upper chamber of the inserts, and DMEM 30% FBS was placed in the lower chambers and incubated for 18 h at 37 °C and 5% CO2. After incubation, the cells were fixed in methanol for 20 min and then stained with Crystal Violet stain solution (#C0121, Beyotime). Five fields per well of invasion cells were examined under a microscope. All experimental assays were performed in triplicates.

### EdU assay

EdU cell proliferation staining was performed using an EdU kit (BeyoClickTM, EDU-488, China). Briefly, the CT26 cells (2 × 10^4^ cells/well) were seeded in 12-well plates and were incubated at 37 °C overnight. The CT26 cells were treated with control (0.1% DMSO), NAP (0.1 μM), TMPs (1 μg/mL), or N_3_-TMPs@NAP (NAP 0.1 μM). Subsequently, the cells were incubated with EdU for 2 h, fixed with 4% paraformaldehyde for 15 min, and then permeated with 0.3% Triton X-100 for another 15 min. The cells were then incubated with the Click Reaction Mixture for 30 min at room temperature in a dark place and incubated with Hoechst 33342 for 10 min.

### Tumor-bearing mouse models

The mouse experiments were approved by the Animal Care Committee of Tongji Medical College, Huazhong University of Science and Technology, China. The right upper limb of BALB/C mice (female, 6 weeks old, Beijing HFK Bioscience Co., Ltd, China) received a subcutaneous injection of CT26 cells (1 × 10^6^) suspended in 100-μL PBS. The mice were ready for experimentation once the tumor size had reached about 5 mm. Liver metastases in the colon cancer models were also prepared as follows. The spleens of 6-week-old BALB/C mice were exposed using laparotomy. Then, the CT26 cells (5 × 10^6^) suspended in 50-μL PBS were injected into their spleens, returned to the abdominal cavity, and sutured the wounds. When the signs of abdominal distension or locomotive deficit appeared or a tumor was detected by palpation, the mice were killed, and their livers and spleens were harvested.

### In vivo* animal PET/CT imaging and biodistribution analysis*

N_3_-TMPs@NAP (200 μg) was infused into the CT26 tumor-bearing mice (n = 3 per group) at the various pre-targeted time points using different delivery methods (tail intravenous or oral administration). ^68^ Ga-L-NETA-DBCO (3.7 MBq) was injected into the mice via their tail veins. The mice were anesthetized with 2% isoflurane, and micro-PET/CT static imaging was performed after 2 h of the injection of ^68^ Ga-L-NETA-DBCO. The static PET/CT images were collected for 10 min using a small-animal PET/CT scanner (Novel Medical, Beijing, China). The mice were sacrificed after PET/CT imaging (n = 3). Their tissues, including brain, heart, lung, liver, spleen, kidney, stomach, small intestine, large intestine, muscle, bone, and tumor tissues, were excised, weighed, and analyzed using a γ-counter. Radioactivity in the organs and tissues was calculated as the percentage of injected dose per gram of tissue (% ID/g) and corrected for radioactive decay. N_3_-TMPs@NAP was incubated with Cy5-NHS for 30 min at 37 °C to form Cy5/N_3_-TMPs@NAP. Cy5/N_3_-TMPs@NAP (100 μg) was infused into the CT26 tumor-bearing mice using different methods (tail intravenous or oral administration). Fluorescence imaging was performed to assess the accumulation of Cy5/N_3_-TMPs@NAP of tumor tissues.

### *Assessment of the *in vivo* antitumor effect*

The CT26 tumor-bearing mice were randomly divided into four groups (n = 5 in each group), which were respectively treated with NS (0.1% DMSO), NAP (20 mg/kg), TMPs (10 mg/kg), and N_3_-TMPs@NAP (NAP 20 mg/kg). After treatments, the tumor sizes and mice body weights were measured every 2 days. On the 14th day, all the mice were sacrificed and their tumor tissues were collected, weighed, photographed, and stored for further histological examinations. Immunohistochemistry was performed to assess the expression levels of proteins Ki67 and CD44 in the tumor tissues. Serums of the NAP and N_3_-TMPs@NAP group was collected for *In-vivo* pharmacokinetic parameters. Briefly, serums were precipitated with acetonitrile at different time points (1, 2, 6, 12, 24, and 48 h) after drug injection, and the supernatant obtained was detected by HPLC.

### In vivo* toxicity studies*

After treatments, the mice’s blood and major organs (liver, spleen, kidneys, heart, and lungs) were also collected. The function indicators of liver and kidney, such as ALT, AST, ALP, BUN, and CRE, were measured using a blood biochemical autoanalyzer (Chemray 240, Rayto Life and Analytical Sciences Co., Ltd, China). The major organs (hearts, livers, spleens, lungs, and kidneys) were stained with H&E and examined under an optical microscope (IX73, Olympus, Japan).

### Transcriptome sequencing

The CT26 cells were treated with NS (0.1% DMSO), NAP (0.2 μM), PBS, TMPs (30 μg/mL). Total RNA was extracted using TRIzol reagent (#15596026, Invitrogen), and transcriptome sequencing was performed by NOVOGENE (Beijing, China) based on the Illumina platform. The prepared libraries were sequenced on an Illumina NovaSeq platform, and 150-bp paired-end reads were generated.

### Western blot analysis

The CT26 and HCT116 cells were harvested and lysed with lysis buffer, containing the phosphatase inhibitors and 1% protease, on ice for 30 min. Then, the cell lysates were centrifuged at 12,000 g for 15 min at 4 °C and the supernatants were collected. The protein concentrations were determined using a protein quantification kit (#P0012S, Beyotime) to ensure that equal amounts of total proteins were loaded into each well of SDS-PAGE gels. The gels were transferred onto PVDF membranes. The membranes were blocked with 5% non-fat milk for 1 h at room temperature and incubated with the primary antibodies overnight at 4 °C. On the second day, the membranes were washed with 1 × TBST for 30 min and incubated with the respective secondary antibodies for 1 h. After incubation, the membranes were washed 3 times with PBS and exposed to X-ray films using ECL detection reagents (#WP20005, Thermo Fisher). The antibodies used in this experiment are as follows; STAT1 antibody (#10144-2-AP, 1:2000), STAT2 antibody (#66485–1-Ig, 1:4000), STAT3 antibody (#10253-2-AP, 1:2000), CD44 antibody (#15675-1-AP, 1:2000), BMI1 antibody (#10832-1-AP, 1:2000), TBK1 antibody(#28397-1-AP, 1:2500) and IRF3 antibody(#11312-1-AP,1:5000) were purchased from Proteintech. GAPDH antibody (#ab8245, 1:3000) was purchased from Abcam. p-TBK1(Ser172) antibody (#5483,1:1000), p-IRF3(Ser386) (#37829, 1:1000) were purchased from Cell Signaling Technology.

### Quantitative RT-qPCR assay

Total RNA was extracted using a Trizol reagent (#15596026, Invitrogen). The extracted RNA samples were reverse-transcribed using a PrimeScriptTM RT reagent Kit (#RR047A, TAKARA, JPN). Quantitative real-time PCR was performed using a TB GreenTM Fast qPCR Mix kit (#RR430A, TAKARA, JPN). GAPDH served as the reference gene, and the 2^−ΔΔCT^ method was used to quantify the fold change in gene expression. The primer sequences for RT-qPCR are provided in Additional file 1: Table S1.

### RNA interference

Gene-specific siRNA were purchased from Sigma-Aldrich. Tumor cells were transfected with siControl or siRNA in Lipofectamine 2000 (#11668019, Thermo Fisher). 12 h after transfection, replace the transfection medium with DMEM containing 10% FBS. siRNA sequences were shown in Additional file 1 (Table S2).

### Chromatin immunoprecipitation (ChIP) and ChIP-qPCR

ChIP was performed using the Chromatin Extraction Kit (#ab117152, Abcam) and ChIP Kit Magnetic-One Step (#ab156907, Abcam), following the manufacturer's instructions. Purified DNA was analyzed using real-time PCR and TB GreenTM Fast qPCR Mix kit (#RR430A, TAKARA, JPN) according to the manufacturer's protocol. Primers used for ChIP-qPCR were shown in Additional file 1: (Table S3).

### Immunohistochemistry

Tumor tissues were fixed with 4% paraformaldehyde, and then dehydrated and embedded in paraffin. From the rehydrated tissue slices, antigen retrieval was performed using the heat-induced antigen retrieval in citrate buffer (Vector Laboratories, CA). The tissues were made permeable with PBS, containing 0.2% TritonX-100, and then treated with 3% hydrogen peroxide to inactivate the endogenous peroxidase. The tissues were washed with PBS, containing 0.05% Tween-20, and then incubated for 2 h with a blocking solution (MOM blocking buffer, Vector Laboratories, CA). The samples were then incubated with CD44 antibody overnight at 4 °C, followed by incubation with secondary antibodies for 1 h at room temperature. Signals were amplified using an ABC kit (Vector Laboratories) and visualized using a 3,3′-diaminobenzidine substrate kit (SK-4105, Vector Laboratories). The tissues were further stained with H&E, dehydrated, and mounted (H5000, Vector Laboratories).

### In vivo* immune response*

Immunofluorescence staining was performed to assess the presence of tumor-infiltrating T cells in the tumor tissues. After treatments, the tumor tissues were collected and incubated with the corresponding antibodies, including CD3 (#17617-1-AP, Proteintech), CD8 (#67786-1-Ig, Proteintech), and CD4 (#66868-1-Ig, Proteintech).

### Bioinformatic analyses

Bioinformatics analyses were performed using the R Bioconductor. DESeq2 package was used for differential analysis and obtaining the average value of different genes. The ggplot2 and pheatmap packages were used to draw volcano maps and heat maps. ClusterProfiler package was used for the gene set enrichment analysis (GSEA). GOplot package was used to draw the Gocircle plot. The protein–protein interaction (PPI) network was constructed using the STRING database (https://cn.string-db.org/). Cytoscape software was employed to visualize the interaction network, which was analyzed using the Mcode algorithm to calculate the interconnected subgraphs of a complex PPI network. The gene expression and clinical data of colon adenocarcinoma were downloaded from the TCGA biolinks packages (version 2.14.1). Ggstatsplot package was used to analyze the correlations between different genes in colon cancer. UCSC Genome Browser was used to visualize the STAT1 ChIP-seq signal profiles in the CD44 gene region. The CD44 promoter sequences and putative STAT1-binding sites were obtained from the Eukaryotic Promoter Database (EPD) (https://epd.epfl.ch//index.php) and (http://jaspar.genereg.net).

### Statistical analyses

Unpaired or paired Student's t-tests were used for group comparisons in statistical analyses, and one-way or two-way ANOVA was used for multiple comparisons. GraphPad Prism 8 was used to evaluate statistical significance (GraphPad Software, Inc.). Statistics were considered significant at P0.05. Every value was expressed in terms of means ± standards.

## Supplementary Information

**Additional file 1: Figure S1. **The diameters of TMPs and N_3_-TMPs@NAP measured by NTA. **Figure S2. **Live and dead cell staining experiments of CT26 cells treated with different methods. Scale bars = 50 μm. **Figure S3. **PET/CT imaging of CT26 tumor-bearing mice after intravenous injection of ^68^Ga-L-NETA-DBCO for 2 h and N_3_-TMPs@NAP (0, 100, 200, 400 μg) for 20 h. **Figure S4.** After oral administration of N_3_-TMPs@NAP (p.o.) for 10 and 20 h as well as intravenous injection of ^68^Ga-L-NETA-DBCO for 2 h, PET/CT imaging of CT26 tumor-bearing mice was performed. **Figure S5.** *In-vivo* pharmacokinetic parameters of NAP and N_3_-TMPs@NAP (n=3). **Figure S6.** Representative H&E staining images of major organs from the euthanized mice. Scale bar = 100 μm. Data are represented as mean ±SD. (n = 5, ***P < 0.001). **Table S1**. The primer sequences for RT-qPCR. **Table S2.** The siRNA sequences. **Table S3.** The primer sequences for ChIP-qPCR.

## Data Availability

The datasets analyzed during the current study are available from the corresponding author upon reasonable request.

## Abbreviations

CSCs: Cancer stem cells
NAP: Napabucasin
TMPs: Tumor-derived microparticles
N_3_: Azidobenzene
PET/CT: Positron emission tomography/computed tomography
NTA: Nanoparticle tracking analysis
NIRF: Near-infrared fluorescence
TEM: Transmission electron microscope
NS: Normal saline
CCK-8: Cell Counting Kit-8
T/M: Tumor to muscle
ALT: Alanine amino transferase
AST: Aspartate aminotransferase
ALP: Alkaline phosphatase
BUN: Blood urea nitrogen
CRE: Creatinine
H&E: Hematoxylin and eosin
EPD: Eukaryotic Promoter Database
GSEA: Gene set enrichment analysis
PPI: Protein–protein interaction
HPLC: High-performance liquid chromatography
