# Supplementary material for: Apoptotic tumor cell-derived microparticles loading Napabucasin inhibit CSCs and synergistic immune therapy
Source: J Nanobiotechnology. 2023 Feb 2;21:37. doi: 10.1186/s12951-023-01792-8 (PMC9893668; doi:10.1186/s12951-023-01792-8)
Supplement: Supplementary file 1 — Additional file 1: Figure S1. The diameters of TMPs and N3-TMPs@NAP measured by NTA. Figure S2. Live and dead cell staining experiments of CT26 cells treated with different methods. Scale bars = 50 μm. Figure S3. PET/CT imaging of CT26 tumor-bearing mice after intravenous injection of 68Ga-L-NETA-DBCO for 2 h and N3-TMPs@NAP (0, 100, 200, 400 μg) for 20 h. Figure S4. After oral administration of N3-TMPs@NAP (p.o.) for 10 and 20 h as well as intravenous injection of 68Ga-L-NETA-DBCO for 2 h, PET/CT imaging of CT26 tumor-bearing mice was performed. Figure S5. In-vivo pharmacokinetic parameters of NAP and N3-TMPs@NAP (n=3). Figure S6. Representative H&E staining images of major organs from the euthanized mice. Scale bar = 100 μm. Data are represented as mean ±SD. (n = 5, ***P < 0.001). Table S1. The primer sequences for RT-qPCR. Table S2. The siRNA sequences. Table S3. The primer sequences for ChIP-qPCR. [file 12951_2023_1792_MOESM1_ESM.docx]

**Title page:**

**Apoptotic tumor cell-derived microparticles loading Napabucasin inhibit CSCs and synergistic immune therapy**

Boping Jing*^1,2,3^*^†^, Feng Guo*^4^*^†^, Yu, Gao*^3,5^*, Yuman Li*^1,2,3^*, Yuji Xie*^1,2,3^*, Yihan Chen*^1,2,3^*, He Li*^1,2,3^*, Tang Gao*^1,2,3^*, Qiaofeng Jin*^1,2,3^*, Li Zhang*^1,2,3,6^*^*^, Mingxing Xie*^1,2,3,6^*^*^.

Affiliations

*^1^*Department of Ultrasound Medicine, Union Hospital, Tongji Medical College, Huazhong University of Science and Technology, Wuhan 430022, China

*^2^*Clinical Research Center for Medical Imaging in Hubei Province, Wuhan 430022, China

*^3^*Hubei Key Laboratory of Molecular Imaging, Wuhan 430022, China

*^4^*Department of Pancreatic Surgery, Union Hospital, Tongji Medical College, Huazhong University of Science and Technology, Wuhan 430022, China

*^5^*Department of Nuclear Medicine, Union Hospital, Tongji Medical College, Huazhong University of Science and Technology, Wuhan 430022, China

*^6^*Shenzhen Huazhong University of Science and Technology Research Institute, Shenzhen 518607, China

^†^ Boping Jing and Feng Guo contributed equally to this work.

***Corresponding Authors:**

Mingxing Xie, No. 1277 Jiefang Ave, Wuhan, Hubei Province 430022, China. Phone: +86-13607108938; Fax: +86-27-85726172. E-mail: xiemx@hust.edu.cn

Li Zhang, No. 1277 Jiefang Ave, Wuhan, Hubei Province 430022, China. Phone: +86-18907131488; Fax: +86-27-85726172. E-mail: [zli429@hust.edu.cn](mailto:zli429@hust.edu.cn)

**Additional file figure**

**Figure S1.** The diameters of TMPs and N_3_-TMPs@NAP measured by NTA.

**
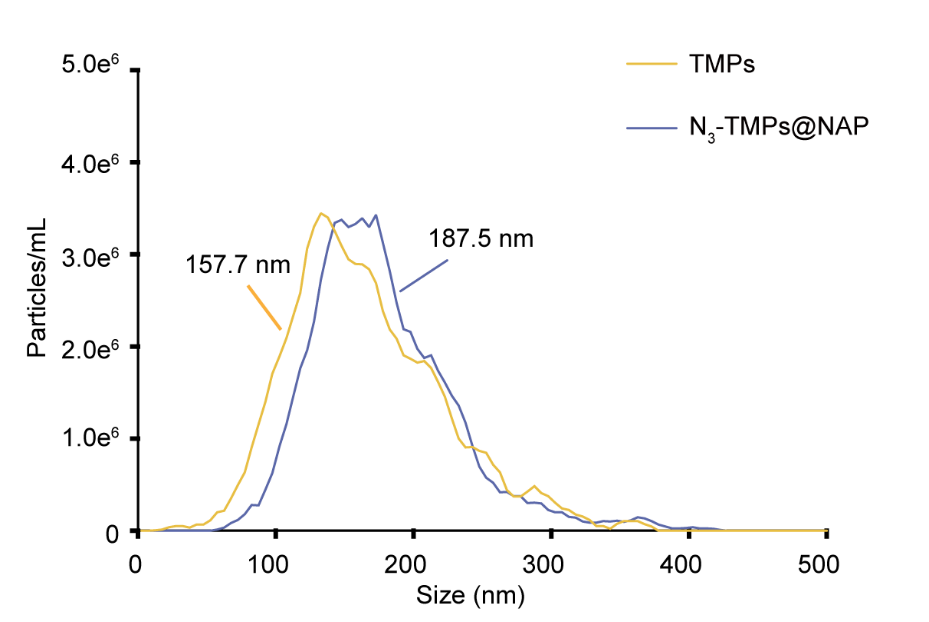
**

**Figure S2.** Live and dead cell staining experiments of CT26 cells treated with different methods. Scale bars = 50 μm.

**
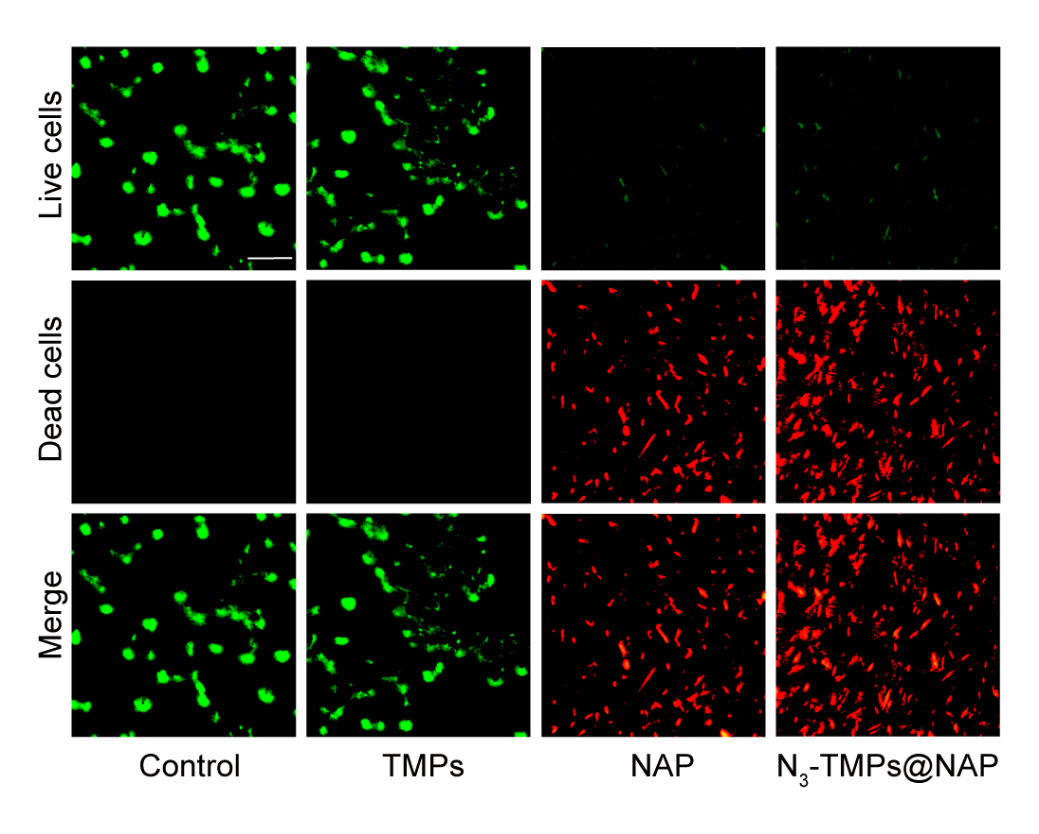
**

**Figure S3.** PET/CT imaging of CT26 tumor-bearing mice after intravenous injection of ^68^Ga-L-NETA-DBCO for 2 h and N_3_-TMPs@NAP (0, 100, 200, 400 μg) for 20 h.

**
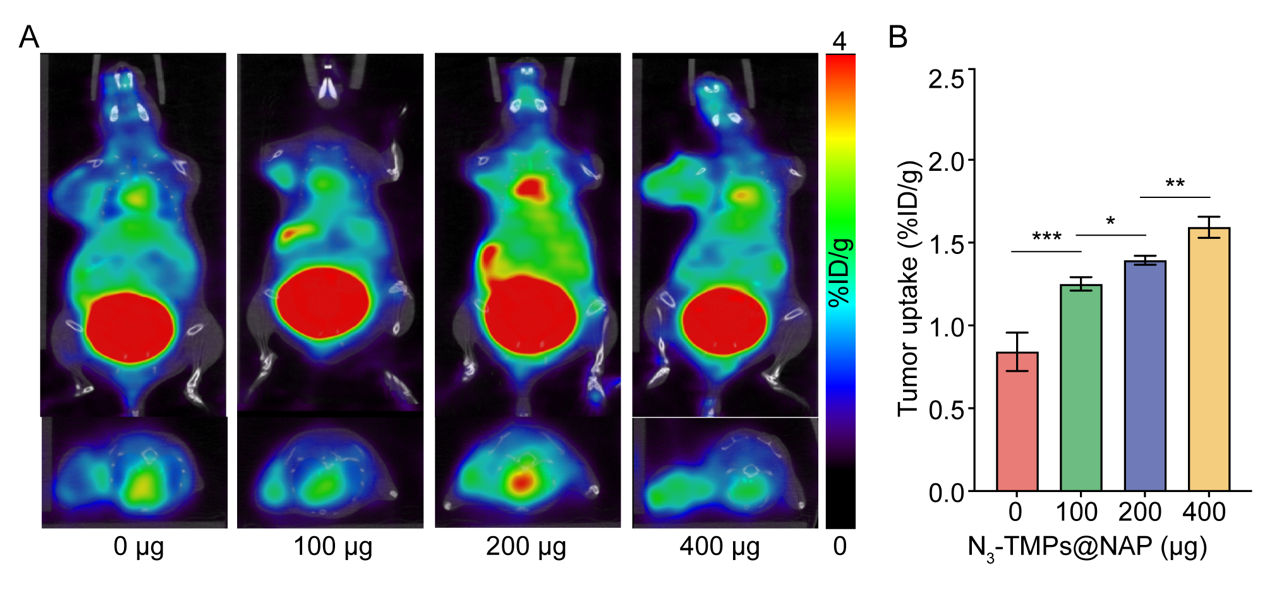
**

**Figure S4. After oral administration of N_3_-TMPs@NAP (p.o.) for 10 and 20 h as well as intravenous injection of ^68^Ga-L-NETA-DBCO for 2 h, PET/CT imaging of CT26 tumor-bearing mice was performed.**

**
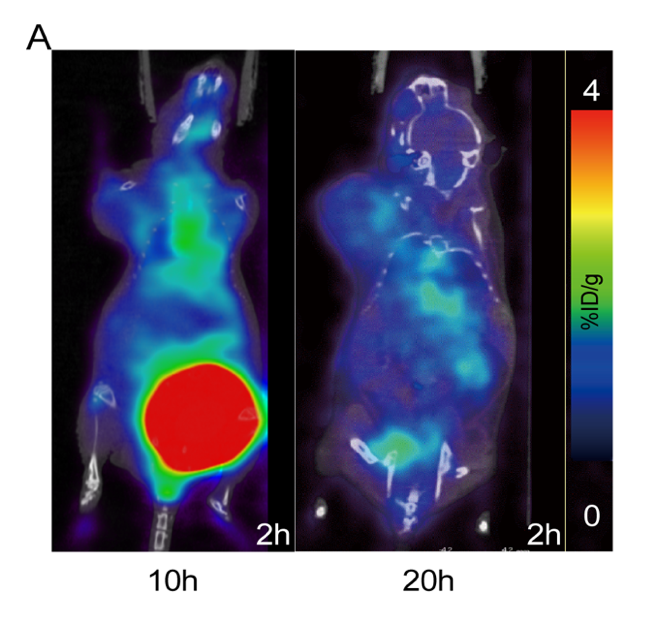
**

**Figure S5.** *In-vivo* pharmacokinetic parameters of NAP and N_3_-TMPs@NAP (n=3).


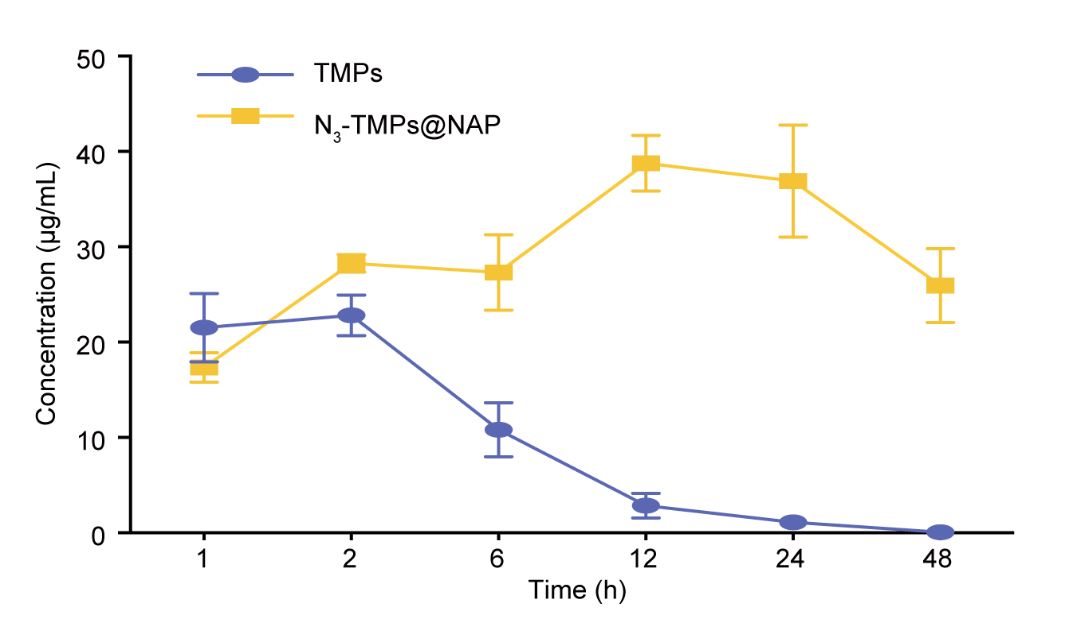


**Figure S6.** **Representative H&E staining images of major organs from the euthanized mice. Scale bar = 100 μm. Data are represented as mean ±SD. (n = 5, ***P < 0.001).**

**
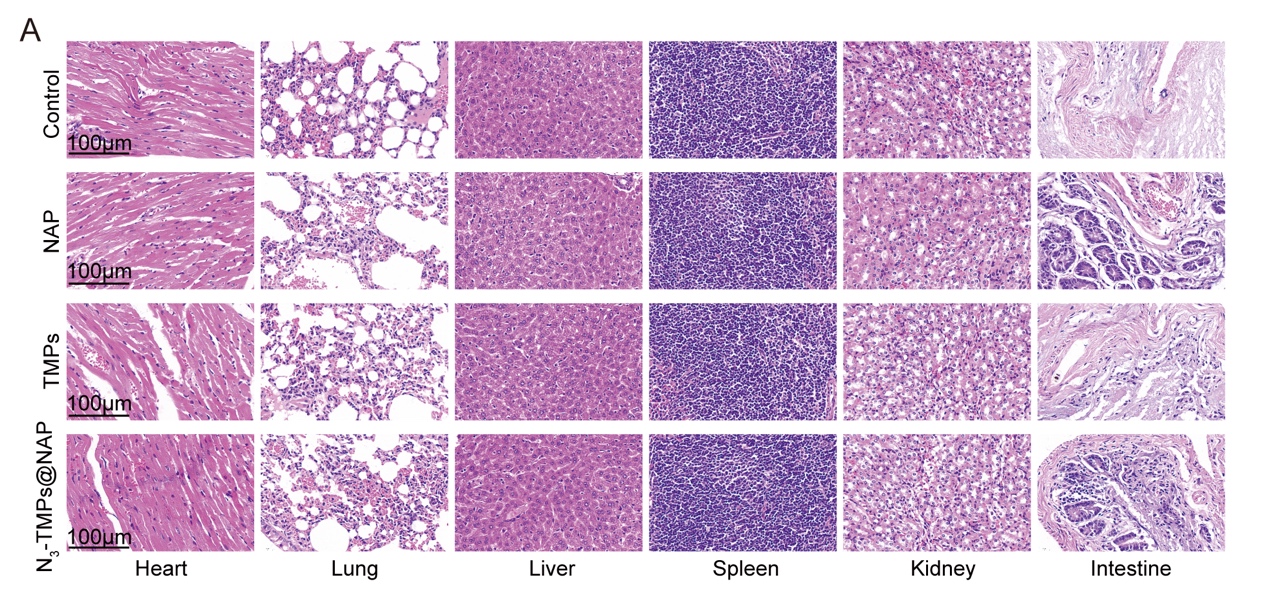
**

**Table S1. The primer sequences for RT-qPCR.**

| Gene | Forward primer (5′ - 3′) | Reverse primer (5′ - 3′) |
| --- | --- | --- |
| CD44 | TCGATTTGAATGTAACCTGCCG | CAGTCCGGGAGATACTGTAGC |
| BMI1 | AAATCCCCACTTAATGTGTGTCC | CTTGCTGGTCTCCAAGTAACG |
| STAT1 | TCACAGTGGTTCGAGCTTCAG | CGAGACATCATAGGCAGCGTG |
| STAT2 | GTTACACCAGGTCTACTCACAGA | TGGTCTTCAATCCAGGTAGCC |
| STAT3 | CACCTTGGATTGAGAGTCAAGAC | AGGAATCGGCTATATTGCTGGT |
| IFNB1 | AGCTCCAAGAAAGGACGAACA | GCCCTGTAGGTGAGGTTGAT |
| ISG15 | GGTGTCCGTGACTAACTCCAT | CTGTACCACTAGCATCACTGTG |
| IL8(CXCL15) | TCGAGACCATTTACTGCAACAG | CATTGCCGGTGGAAATTCCTT |
| GAPDH | AGGTCGGTGTGAACGGATTTG | GGGGTCGTTGATGGCAACA |

**Table S2. The siRNA sequences.**

| siSTAT1#1(mouse) | AAAGCAAGCGTAATCTCCAGGATAA |
| --- | --- |
| siSTAT1#2(mouse) | CAGATGTCCATGATCATCTACAACT |
| siSTAT1#1(human) | AAAGCAAGCGTAATCTTCAGGATAA |
| siSTAT1#2(human) | CAGGAAGACCCAATCCAGATGTCTA |

**Table S3. The primer sequences for ChIP-qPCR.**

| Gene | Forward primer (5′ - 3′) | Reverse primer (5′ - 3′) |
| --- | --- | --- |
| CD44 primer1 | TAGTCACAGCCCCCTCGCTT | ACCGCTTCGGAAGTTGGCTG |
| CD44 primer2 | ACTGAGAGGGGCGAGGTCTT | TTCTCCAGGGCCACTCCACA |
